# Prevalence of domestic violence and associated factors among pregnant women attending antenatal care service at University of Gondar Referral Hospital, Northwest Ethiopia

**DOI:** 10.1186/s12905-018-0632-y

**Published:** 2018-08-14

**Authors:** Elfalet Fekadu, Getachew Yigzaw, Kassahun Alemu Gelaye, Tadesse Awoke Ayele, Tameru Minwuye, Tinsae Geneta, Destaw Fetene Teshome

**Affiliations:** 1 Department of Gynecology and Obstetrics, School of Medicine, College of Medicine and Health Sciences, University of Gondar, Gondar, Ethiopia; 2 Department of Epidemiology and Biostatistics, Institute of Public Health, College of Medicine and Health Sciences, University of Gondar, Gondar, Ethiopia; 3 Dabat Research Centre Health and Demographic Surveillance System, Institute of Public Health College of Medicine and Health Science, University of Gondar, Gondar, Ethiopia

**Keywords:** Domestic violence, Emotional violence, Physical violence, Sexual violence

## Abstract

**Background:**

Domestic violence during pregnancy with its many negative fetal and maternal outcomes is a common public health problem all over the world. Nonetheless, the problem is not well investigated and understood in Ethiopia. Hence, this study aimed to assess the prevalence of domestic violence and associated factors among pregnant women attending the University of Gondar Referral Hospital antenatal care (ANC) services.

**Methods:**

A hospital-based cross-sectional study was conducted from March–May 2016. A total of 450 pregnant women who visited the clinic were included in the study. A systematic random sampling technique was used to select study participants, and a pretested structured questionnaire was employed to collect data. The WHO multi-country study on women’s health and domestic violence against women was used to assess the violence against pregnant women. Descriptive statistics such as means, frequencies and percentages were computed. A multivariable logistic regression analysis was carried out to identify factors associated with domestic violence, and variables with *p*-values < 0.05 were considered as statistically significant.

**Results:**

Of the total pregnant women surveyed, 58.7% were victims of at least one form of domestic violence during pregnancy, emotional violence being the most common (57.8%). The multivariable logistic regression analysis showed that house wives (adjusted odd ratio (AOR) = 3.43, 95% CI: 1.63, 7.21), women with no salary of their own (AOR = 3.37, 95% CI: 2.14, 7.95), partners’ daily use of alcohol (AOR = 4.59, 95%CI: 1.82, 11.56), women who believed in women’s rights to decide to be pregnant (AOR = 1.77, 95%CI: 1.18, 2.89), and women who disobeyed their partner (AOR = 2.36, 95%CI: 1.37, 4.07) were found to be positively and significantly associated with domestic violence during pregnancy.

**Conclusion:**

A high proportion of pregnant women experienced domestic violence during their pregnancy. Being a housewife, poor income status, partners’ use of alcohol, unwanted pregnancy, and disobeying of the women to their partner were factors associated with domestic violence during pregnancy. Evidence based female empowerment, especially the empowerment of women without income of their own, partner education and positive relations between partners are very important to minimize the problem.

## Background

Violence against women committed by intimate partners is an important public health and human rights issue [1]. Domestic violence against women is a common practice in all countries of the world though the prevalence varies from country to country [2]. According to the World Health Organization (WHO) multi-country study, the problem ranges from 15% in Japan to 71% in rural Ethiopia [3].

More commonly, pregnancy is considered to be a critical time at which domestic violence begins or amplifies due to women’s increased physical and mental vulnerability [4]. Studies conducted in Kisumu district hospital, Kenya [5], Rwanda [6], Kwazulu-Natal, South Africa [7], and Sao Paulo, Brazil [8], showed that one in three pregnant women experienced either physical, emotional, sexual violence or both by intimate partners during pregnancy.

Domestic violence can lead to poor physical [9, 10], mental [9, 11–14], sexual and reproductive health outcomes [10]. Violence during pregnancy is particularly harmful as it threatens both maternal and fetal life. Previous studies revealed that pregnant women who experienced violence were found to have increased risks of ante-partum hemorrhage [15], intrauterine growth retardation [16], premature rupture of membrane [17, 18], cesarean delivery [18–20], preterm birth [21, 22], low birth weight babies [17, 18, 21, 23], stillbirth [15, 23, 24], and neonatal mortality [24, 25] when compared with those who did not experience the problem.

Despite this substantial health burden, previous studies focused on the prevalence and determinants of domestic violence against women; so little is known about the extent of violence on the most at risk pregnant women in Ethiopia as well in the study area. Moreover, evidence is unavailable on what factors put pregnant women at increased risk of domestic violence during their pregnancy. Hence, this study aimed to assess the prevalence of domestic violence against women and associated factors among pregnant women visiting the University of Gondar Referral Hospital for ANC services. The findings are expected to increase the awareness of health care providers working with pregnant women in screening, detecting and readily supporting pregnancies vulnerable to domestic violence. The information will also help health programmers and policy makers at large to design preventive and controlling strategies to alleviate the problem.

## Methods

### Study design and period

An institution-based cross-sectional study was conducted from March to May 2016 to determine the prevalence of domestic violence and its associated factors among pregnant women visiting the University of Gondar Referral Hospital for antenatal care services.

### Study area

The hospital is found in the ancient and historic town of Gondar, northwest Ethiopia, 741 km from Addis Ababa. It is one of the biggest tertiary level referral and teaching hospitals in the Amhara Regional State and provides promotive, preventive and curative services to over 5 million inhabitants in the catchment area. The hospital consists of 4 operating rooms, 4 intensive care units, and 13 wards with 327 beds. The antenatal care clinic is one of the departments which provide services to 50–70 pregnant women coming from Gondar town and the nearby districts per day. The hospital also serves as a research center and provides practical training to medicine and health science students.

### Study population

All pregnant women who attended antenatal care services in the University of Gondar Referral Hospital were the source population. Such women aged 18 to 49 years and attended the Antenatal Care clinic in the hospital during the data collection period were included in the study. Pregnant women on labor and had danger signs of pregnancy were excluded.

### Sample size determination and sampling procedures

To determine the sample size, we used the single population proportion formula and the assumption that the percentage of pregnant women facing domestic violence was 50%, 95% confidence interval (CI), 4% of precision level, and non response rate of 10%. Thus, the total sample was 450 pregnant women. First ANC service reports of the previous 3 months were reviewed, and the average number of pregnant women who visited the ANC Clinic per month was estimated at 1320. A systematic random sampling technique was used to select study participants. The first participant was selected using the lottery method, and every 10th pregnant women was chosen based on their visiting order till the sample size was met.

### Data collection tools and procedures

A pretested structured questionnaire consisting of sociodemographic and economic characteristics, attitudes towards women’s role and the extent of property ownership in their life, sociodemographic and behavioral factors of partners/husbands were used to collect the data.

The WHO multi-country study on women’s health and domestic violence against women was used to assess domestic violence against pregnant women [3]. The response to each item was either Yes or No. Accordingly, participants who respond “Yes” to any of the five questions on physical intimate partner violence and/or three questions on sexual intimate partner violence, and/or four questions on emotional intimate partner violence during pregnancy was considered as incident cases of domestic violence victimization.

The questionnaire was first prepared in English and translated into Amharic (local language) by language experts and retranslated to English to check for consistency. The questionnaire was pretested on 5% of the study participants in similar setting, and amendments were made accordingly. Four midwives trained for two days collected the data. The investigator and a supervisor oversaw the data collection process and made corrections daily.

### Operational definitions

**Physical violence** was presumed to have taken place when a woman/participant provided “Yes” answers to the 5 questions that inquired her whether she was 1) thrown at something that could hurt, 2) pushed or shoved, 3) hit with the fist or something else that could hurt, 4) kicked, dragged, beaten up, choked or burned on purpose; and 5) if a gun, knife or any other weapon was used against her.

**Sexual violence** was presumed to have taken place when a woman was physically forced to have sexual intercourse against one’s will, or having sexual intercourse because of being afraid of what a partner might do, or being forced to do something sexual one has found degrading or humiliating.

**Emotional violence** was defined as being insulted or made to feel bad about one-self, humiliated or be little in front of others, intimidated or scared on purpose (for example by a partner yelling and smashing things), or threatened with harm (directly or indirectly in the form of a threat to hurt someone the respondent cares about).

### Statistical analysis

The data was checked for completeness, coded and entered into EPI INFO version 7 and exported to SPSS 20 for analysis. Means, frequencies, and percentages were used to summarize data and texts and tables to present data. Bivariate analysis was done to see the associations of each independent variable with domestic violence during pregnancy. Variables which had *p* values up to 0.2 were considered for the multivariable logistic regression analysis to control the effects of confounding variables. The Hosmer-Lemeshow goodness of fit test was checked for the model. Finally, variables which had significant associations with domestic violence during pregnancy were identified on the basis of OR with 95% CI and *p*-value < 0.05.

## Results

### Participants’ characteristics

A total of 450 pregnant women participated in the study with a response rate of 100%. The mean age of the participants was 27 (SD±4.5) years; 415(92.2%) of the participants were Amhara by ethnic origin, 352 (78.2%) were Orthodox Christians, and 393 (87.3%) were married. Three hundred seventy three (82.9%) of the participants were urban dwellers, 220 (48.9%) housewives, and 125 (27.8%) had higher education. Regarding their economic status, 252 (56.0%) had no own salary, 75(16.2%) were worrying about food supply at home last month, and 47.1% faced difficulty finding 50 Ethiopian Birr (ETB) for emergency (Table 1).

**Table 1:** Socio demographic characteristics of pregnant mothers at University of Gondar referral hospital ANC, northwest Ethiopia, 2016

Characteristics	Frequency	Percent
Age		
18–24	159	35.3
25–30	209	46.4
31–35	61	13.6
36–40	20	4.4
40–49	1	0.2
Ethnicity		
Amhara	415	92.2
Tigre	26	5.8
Others*	9	2.0
Religion		
Orthodox	352	78.2
Muslim	79	17.6
Others**	19	4.2
Marital status		
Single	32	7.1
Married	393	87.3
Widowed	10	2.2
Divorced	15	3.3
Educational level		
Illiterate	72	16.0
Completed 1–6 grade	48	10.7
Completed 7–12 grade	205	45.6
College and above	125	27.8
Occupation		
Housewife	213	47.3
Student	18	4.0
Merchant	46	10.2
Farmer	28	6.2
Government employee	145	32.2
Income status, ETB		
No salary	252	56.0
< 500	31	6.9
501–1500	50	11.1
1501–3000	77	17.1
> 3000	40	8.9
Residence		
Urban	373	82.9
Rural	77	17.1

*Oromo, Bete Israel **Protestant, Catholic

### Partners’ sociodemographic characteristics

Of the partners, 198 (44%) were between 25 to 30 years of age; 230(51.1%) were more educated than their wives, and 312(69.3%) were employed (Table 2).

**Table 2:** Partners’ characteristics of pregnant women attending ANC at University of Gondar referral hospital, northwest Ethiopia, 2016

Characteristics	Frequency	Percent
Age, in years		
18–24	17	3.8
25–30	198	44.0
31–35	69	15.3
36–40	96	21.3
> 40	70	15.6
Occupation		
Unemployed	108	24.0
Farmer	30	6.7
Employed	312	69.3
Educational status		
Less educated than his wife	76	16.9
Same level of education	144	32.0
More educated than his wife	230	51.1
Use of alcohol		
Daily	60	13.3
Sometimes	222	49.3
Never	168	37.3
Use of drug		
Daily	16	3.6
1–2 per week	31	6.9
1–3 times per month	22	4.9
Never	381	84.7

### Partners’ behavioral characteristics

One hundred sixty-eight (37.3%) of the participants partners never used alcohol, 60 (13.3%) drank alcohol daily, 381(84.7%) never took any type of drug, and only 16 (3.6%) used drug/medications on daily basis.

### Attitudes of the participants about women’s role and the extent of ownership in their life

One hundred ten (24.4%) of the participants believed that women’s role in life was to cook and take care of the home; 178(39.6%) held women should tolerate violence to keep their family together, and 44 (9.8%) conceived that sometimes wives need to be beaten. Of the participants, 328 (72.9%) stated that a husband should own his wife, 354 (78.7%) said she should obey her husband or partner, and if not, 238(52.9%) stated a wife should be punished by her husband. Some, 146(32.4%) believed that final decisions at home should be made by the husband; 62 (13.8%) stated a man should use force to keep his reputation if he had to, and 81 (18%) believed physical violence was a sign of love. Two hundred eighty-seven (63.8%) of the participants reported that a wife could not refuse to have sex in a marriage (Table 3).

**Table 3:** Attitudes of the study participant about woman’s role in the relationship and the extent of ownership in their life

Variables	Numbers	Percent
Woman’s role in life is to cook & take care of her home	110	24.4
Woman need to be beaten sometimes	44	9.8
It is a woman’s responsibility to decide getting pregnant	142	31.6
Woman should tolerate violence to keep her family together	178	39.6
Man should use force to keep his reputation if he has to	92	20.4
Man should make final decision at home	146	32.4
Woman should obey her husband	350	77.8
Woman can’t refuse sex in marriage	287	63.8
Biting is a sign of love	81	18.0
Wife should be punished by her husband	238	52.9
Husband owns his wife	328	72.9

### Domestic violence against pregnant women

The overall prevalence of domestic violence among pregnant woman in this study was 58.7% (95% CI: 53.8, 63.1), emotional violence being the most common (57.8%), followed by physical (32.2%), and sexual (7.6%). A high prevalence of domestic violence (53.8%) was observed among pregnant women with no income of their own, followed by housewives (52.3%).

### Factors associated with domestic violence during pregnancy

In the multivariable logistic regression analysis, variables such as women’s occupation, income, partners’ use of alcohol, women’s responsibility to be pregnant, and obey their partners remained significant predictors of domestic violence during pregnancy after adjustments for the possible effects of confounders.

Hence, housewives were three times (AOR = 3.43, 95% CI: 1.63, 7.21) as likely to experience domestic violence during pregnancy compared with employed women. The likelihood of domestic violence during pregnancy among pregnant women with no salary of their own was found to be three times (AOR = 3.37, 95% CI: 2.14, 7.95) as likely compared to pregnant women with their own income/salary. The likelihood of domestic violence among pregnant women whose husbands used alcohol daily were five times (AOR = 4.59, 95% CI: 1.82, 11.56) as likely compared to women whose husbands never used alcohol. The likelihood of domestic violence among pregnant women who believed the responsibility of deciding pregnancy to be women’s was two times (AOR = 1.77, 95% CI: 1.18, 2.89) as compared to women who didn’t believes. A woman who didn’t obey her husband was two times (AOR = 2.36, 95% CI: 1.37, 4.07) as likely to face domestic violence compared to a woman who obeyed (Table 4).

**Table 4:** Bivariable and multivariable binary logistic regression analysis of factors associated with domestic violence among pregnant women at University of Gondar referral hospital, northwest Ethiopia, 2016

Variable	Domestic Violence		COR (95% CI)	AOR (95% CI)
	Yes	No		
Mothers’ Educational status				
Illiterate	49	23	2.03 (1.11, 3.73)	0.83 (0.35, 1.99)
Completed 1–6 grade	34	14	2.32 (1.13, 4.73)	1.20 (0.48, 1.98)
Completed 7–12 grade	117	88	1.27 (0.81, 1.98)	0.81 (0.46, 1.44)
College and above	64	61	1	1
Occupation				
House wife	138	75	2.33 (1.51, 3.59)	3.43 (1.63, 7.21)*
Student	10	8	1.58 (0.59, 4.24)	1.09 (0.31, 3.85)
Merchant	38	8	6.01 (2.62, 13.79)	6.53 (2.57, 16.62)*
Farmer	14	14	1.27 (0.56, 2.85)	0.83 (0.25, 2.83)
Government employee	64	81	1	1
Mother’s personal income, ETB				
No salary	142	110	0.55 (0.27, 1.14)	3.37 (2.14, 7.95)*
< 500	25	6	1.79 (0.58, 5.47)	1.99 (0.56, 7.04)
500–1500	27	23	0.50 (0.21, 1.21)	0.47 (0.17, 1.32)
1501–3000	42	35	0.51 (0.23, 1.59)	0.61 (0.24, 1.57)
> 3000	28	12	1	1
Partners’ occupation				
Unemployed	61	47	0.96 (0.62, 1.50)	1.01 (0.62, 1.65)
Farmer	24	6	2.97 (1.18, 7.48)	1.66 (0.55, 4.96)
Employed	179	133	1	1
Partners use of alcohol				
Never	95	73	1	1
Sometimes	116	106	0.84 (0.56, 1.26)	0.86 (0.54, 1.36)
Daily	53	7	5.82 (2.50, 13.55)	4.59 (1.82, 11.56)*
Woman need to be beaten sometimes				
Yes	35	9	3.01 (1.41, 6.42)	2.32 (0.92, 5.87)
No	229	177	1	1
It is a woman’s responsibility to decide getting pregnant				
Yes	99	43	1.99 (1.31, 3.04)	1.77 (1.18, 2.89)*
No	165	143	1	1
Woman should obey her husband				
Yes	192	158	1	1
No	72	28	2.12 (1.30, 3.44)	2.36 (1.37, 4.07)**

*P value< 0.05

## Discussion

This is one of the first studies reporting the prevalence of domestic violence among pregnant women during pregnancy in Ethiopia, particularly in the study area (Gondar and its surroundings). The study found that over half (58.7%) of pregnant women suffered domestic violence by an intimate partner during pregnancy. Emotional violence was the most common, followed by physical and sexual violence. The finding is consistent with that of study conducted among native Americans (52.5%) [26]. Our finding is significantly higher than those of studies conducted in Kisumu district hospital, Kenya (37%) [5], Rwanda(35.1%) [6], Sao Paulo, Brazil(34.6%) [8], KwaZulu-Natal, South Africa(31%) [7], Mulago Hospital, Uganda(27.7%) [17], Lima, Peru (45.1%) [27], Portuguese health institutions (43.4%) [28], Pakistan (51%) [29], Hull Maternity Hospital, UK (17%) [30]. It is also higher than that of a systematic review of African studies conducted between 2000 and 2010 and found that the prevalence of intimate partner violence ranged from 2 to 57% with an overall prevalence of 15.23% [31]. This difference might be due to differences in sampling techniques to recruit study participants. For instance, studies in Rwanda and Portugal used a convenient sampling technique which might have led to a lower proportion of domestic violence because volunteers are different from non-volunteers. The outcome assessment tool might also be the possible reason for the differences in the prevalence of domestic violence. Studies conducted in Uganda and UK used a woman abuse screening tool, whereas this study used the WHO tool to assess the outcome variable. The other possible explanation for the difference might be the type of data collection technique (self-administered versus interview) the researchers used and the study settings.

However, the current finding is lower than that of a study conducted in southern Appalachia (81%) [32]. The possible reason for the difference is that the former work was conducted on a smaller sample (104) and only pregnant women in the lower socioeconomic status, resulting in the over estimation of the prevalence of the problem.

Like other studies conducted in different parts of the country, this work identified factors associated with domestic violence during pregnancy. Women’s occupation was one of the factors associated with the violence. Thus, the likelihood of domestic violence during pregnancy was three times higher among housewives compared to employed women. The possible explanations could be the fact that most housewives in this study didn’t complete higher education and stayed at home carrying out domestic activities which made them more dependent on their husbands. This is evidenced by the fact that women with no education are four times as likely to face violence during pregnancy as women with more than secondary education [33]. As long as the husband is the family financial source, housewives remain more vulnerable, less autonomous, and economically more dependent on their husbands, which results in disagreements and different forms of violence.

Women’s personal income was one of the factors influencing domestic violence during pregnancy in this study. Women with no salary of their own were three times as likely to experience domestic violence during pregnancy compared with pregnant women who had their own earnings. This finding is supported by those of studies conducted in Awi zone, Ethiopia [34], rural Bangladesh [35], Lima, Peru [27]. The possible reason is that women lacking own income might be exposed to specific stresses, frustration, and a sense of inadequacy for having failed to live with their partners. This might in turn lead to marital disagreements or poor relationships, making it more difficult for women to avoid violent [36].

The likelihood of domestic violence during pregnancy was five times higher among women whose husbands took alcohol daily compared to those who never consumed alcohol. The finding is consistent with those of studies conducted in Gondar [37], western Ethiopia [38], Awi zone, Ethiopia [34], Shimelba, Eritreans’ refugee camp, Ethiopia [39], Kisumu district hospital, Kenya [5], Rwanda [6], Sao Paulo, Brazil [8], the 9 countries of the WHO multi-country study, including Ethiopia [40]. This also was a determinant variable that increased the risk of domestic violence during pregnancy in the systematic reviews of 19 African journals(2000-2010GC) [31]. This is due to the fact that alcohol consumption directly affects the consumers’ cognitive (the ability to perceive, integrate, and process information) and physical functions. This distortion in thinking might cause the users to behave aggressive in the relationship, increase the sense of power and control, leading to exercising the power and control on partners [41].

The likelihood of domestic violence on pregnant women who believed pregnancy decision was the responsibility of women was two times as likely compared to women who didn’t believes. This is in line with the results of studies conducted in Mulago hospital, Uganda [17], Sao Paulo, Brazil [8], Portuguese health institutions [28], Pakistan [29], and South Lebanon [42]. Evidence suggests that pregnancy should be decided by both partners’ willingness and participation. But the mismatch of intentions and decisions to have children might result in harsh relationships between partners, resulting in further emotional and physical violence on female partners.

Women who didn’t obey their husbands were two times more likely to face domestic violence during pregnancy than women who did. This is due to the fact that in Ethiopia there is a deep rooted belief that women should obey and serve their husbands even though it is not supported by law. It is usual that if women didn’t obey their husbands, they should be punished by their husbands by being stubbed, boiled, and kicked.

The first strength of this work is the authors’ use of an adopted standard and a validated instrument of WHO multi-country study on women’s health and domestic violence against women. Its second merit is the adequacy of the training given to data collectors and the pretesting of the tools in an appropriate setting. On the other hand, cross-sectional nature of the work made the determinations of the directions of associations between variables difficult is one of its limitations. Besides, the fact that the data collection technique was not qualitative might have limited the richness and the integrity of the data.

## Conclusion

The prevalence of domestic violence among pregnant woman visiting the University of Gondar Referral Hospital for antenatal care services was high. Being housewives, lack of own salary, partners’ daily consumption of alcohol, mothers’ refusal to obey, pregnancy decisions were positively and significantly associated with domestic violence during pregnancy. Evidence based women empowerment, especially the empowerment of women with no salary of their own, partners’ education, and building positive relations between partners is very important to minimize violence during pregnancy.

## Abbreviations

ANC: Antenatal care service
AOR: Adjusted odd ratio
DV: Domestic violence
ETB: Ethiopian birr
IUGR: Intrauterine growth restriction
UOG: University Of Gondar
WHO: World Health Organization
